# Behavioral health risk factor profiles in general hospital patients: identifying the need for screening and brief intervention

**DOI:** 10.1186/s12889-019-7931-6

**Published:** 2019-11-29

**Authors:** Jennis Freyer-Adam, Florian Noetzel, Sophie Baumann, Ali Alexander Aghdassi, Ulrike Siewert-Markus, Beate Gaertner, Ulrich John

**Affiliations:** 1 Institute for Medical Psychology, University Medicine Greifswald, Walther-Rathenau-Str. 48, 17475 Greifswald, Germany; 2 German Center for Cardiovascular Research, Site Greifswald, Fleischmannstr. 42-44, 17475 Greifswald, Germany; 3 Clinic and Policlinic of Urology, University Medicine Greifswald, Ferdinand-Sauerbruch-Straße, 17475 Greifswald, Germany; 4 Institute of Social Medicine and Prevention, University Medicine Greifswald, Walther-Rathenau-Str. 48, 17475 Greifswald, Germany; 5 Institute and Policlinic of Occupational and Social Medicine, Faculty of Medicine, Technische Universität Dresden, Fetscherstraße 74, 01307 Dresden, Germany; 6 Department of Internal Medicine A, University Medicine Greifswald, Ferdinand-Sauerbruch-Straße, 17475 Greifswald, Germany; 7 Department of Epidemiology and Health Monitoring, Robert Koch Institute Berlin, General-Pape-Str. 62-66, 12101 Berlin, Germany

**Keywords:** Risk factors, Health behaviors, Smoking, Alcohol, Physical inactivity, Overweight, General hospital, Health care, Prevention, Chronic diseases

## Abstract

**Background:**

Little is known about the clustering of behavioral health risk factors (HRFs), namely the occurrence of 16 specific combinations of tobacco smoking, at-risk alcohol use, overweight and physical inactivity in general hospital patients. Furthermore, social inequalities in HRFs, health and life expectancy are a major concern in public health. In order to establish the need for screening and intervention in general hospital care, the study aimed to determine the co-occurrence of HRFs in patients in four medical departments, and to investigate differences by gender, age and socio-economic characteristics.

**Methods:**

Over 17 months, a systematic multiple HRF screening was conducted at one general hospital in northeastern Germany. In total, 6251 18–64 year old patients (92% of eligibles) participated. Proportions and confidence intervals were calculated for all 16 HRF profiles stratified by department, gender, age group, school education, and employment status.

**Results:**

In total, 92.2% of the participants (58.6% male) reported ≥1 HRF, and 65.7% ≥2 HRFs. Men (71.2%), patients aged 35–49 (67.9%) and 50–64 years (69.5%), lower educated (79.0%), and unemployed (77.8%) patients had larger proportions of ≥2 HRFs than their counterparts. In all departments, the most common HRF profiles included overweight. HRF profiles that included alcohol and/ or smoking were more common in ear-nose-throat and trauma surgery than in internal medicine and general surgery patients. Men had higher rates concerning almost all HRF profiles including ≥2 HRFs and alcohol; women concerning profiles that included ≤2 HRFs and inactivity. In older patients, profiles with ≥2 HRFs including overweight; and in younger patients, profiles with smoking and/or alcohol were more common. In lower educated patients, profiles with ≥2 HRFs including inactivity; and in higher educated patients profiles with ≤2 HRFs including alcohol were more common. Compared to others, unemployed patients had higher rates of profiles with ≥3 HRFs including smoking.

**Conclusions:**

Two in three patients require interventions targeting two or more HRFs. The findings help to develop screening and brief intervention for patients with specific health risk profiles, that can reach most patients, including those most in need and those most hard to reach, with socio-economically disadvantaged people in particular.

**Registry:**

clinicaltrials.gov: NCT01291693.

## Introduction

Modifiable behavioral health risk factors (HRFs), particularly tobacco smoking, at-risk alcohol use, unbalanced diet and physical inactivity, are major contributors to the development of non-communicable diseases and to all-cause deaths [1–3]. HRFs inhibit successful recovery or improved wellbeing and increase mortality after diagnosis as found for cancer patients (e.g. [4, 5]). To prevent and treat prevalent non-communicable diseases, abstaining from tobacco smoking, keeping body weight within the healthy range, being physically active as part of everyday life, and limiting alcohol consumption is recommended [2, 6–8].

The energy-balance related HRFs, i.e. physical inactivity and either unhealthy diet or overweight; as well as the two substance-use related HRFs smoking and at-risk alcohol use are intertwined and often cluster [9, 10]. However, a total of 16 specific combinations of the four major modifiable HRFs are possible and observed in the general population [11–13]. About half of the general population in high-income countries report multiple, i.e. two or more of the four HRFs; and even larger proportions are found when insufficient vegetable and fruit intake is considered as an indicator of unhealthy diet instead of overweight [11–14]. The EPIC-Norfolk prospective population study revealed strong trends of increasing mortality with an increasing number of HRFs, particularly cardiovascular causes of death [15]. A protective effect on mortality risk was found with each additional health recommendation met [16]. Furthermore, co-occurring HRFs may not only have an additive but more than a multiplicative effect on disease incidence and/ or mortality as was found for example for alcohol and smoking concerning various cancers [17, 18].

Gender, age and socio-economic status (SES) are related to the accumulation of HRFs and HRF profiles. A systematic review revealed a greater number of HRFs in men than in women, while findings concerning age were rather mixed with some studies showing more HRFs among younger and other studies showing more HRFs among older people [19]. More risky HRF clusters were also found in people with lower levels of education [19]. Social inequalities in life expectancy and health between persons at the bottom and those at the top of the social scale are a major concern in public health, and the accumulation of HRFs such as alcohol and smoking has been suggested to explain these differences [13, 20–22]. Recent findings suggest that SES may be even more relevant than gender in the accumulation of the four HRFs [23].

To address behavioral HRFs in people, the general hospital has been found to be particularly suitable. Hospitalization itself may be a health event that might motivate individuals to change unhealthy behaviors [24, 25]. Increased patient motivation to change HRFs was found for smokers and alcohol dependent patients [24, 26, 27], and could provide a “window of opportunity” for brief behavior change interventions [26]. Among a sample of adult general hospital patients in the United Kingdom who participated in a survey post-discharge, more than 80% considered the hospital a good location for receiving health education on the HRFs, and agreed that all patients should be asked about HRFs [28]. Brief interventions developed to be implemented in health care often target single HRFs. They have been found to be effective in altering single behaviors [29–37] and in improving measures of health [31, 38]. However, interventions targeting multiple HRFs may be more cost-efficient and effective in preventing or treating non-communicable diseases as various interdependent issues may be addressed (e.g. [19, 39]). Encouraging findings on the efficacy of such interventions applied in the general population and general practice setting have been reported [40–43].

Given the significance of HRFs in the development of chronic diseases [1], HRF profiles including either one or more of the four HRFs might be expected to be particularly common in general hospital inpatients. However, systematically drawn data is scarce. A systematic screening study that assessed five behavioral HRFs (the four plus low vegetable and fruit intake) and depressive symptoms at three sites in Germany revealed that 83% of the patients screened positive for two or more of the six screening targets [44]. HRF profiles and their variation across gender, age and SES have not been investigated in the general hospital. Furthermore, little is known about whether hospital departments vary in occurrence of multiple HRFs in their patients, and whether different screening and intervention foci may be justified or needed to address different HRFs or HRF profiles.

The aims of the study were: 1. To determine the co-occurrence of four behavioral HRFs (i.e. tobacco smoking, at-risk alcohol use, overweight, physical inactivity) and their co-occurrence in 16 HRF profiles among patients attending a general hospital. 2. To describe and to compare HRF profiles across four major medical departments (i.e. general medicine, general surgery, trauma surgery, ear-nose-throat). 3. To describe and compare HRF profiles by gender, age and variables related to SES (i.e. school education, employment status). The results will be useful to show the need for interventions targeting multiple HRFs, and to design a comprehensive screening and intervention protocol.

## Methods

Data were obtained as part of the screening procedure for the randomized controlled trial “Testing delivery channels of individualized motivationally tailored alcohol interventions among general hospital patients: in-person versus computer-based, PECO” (ClinicalTrials.gov: NCT01291693). As described in more detail elsewhere [45, 46], the trial tested the comparative two-year efficacy of two ways of delivering brief interventions, namely in-person counseling and computer-generated written feedback, targeting the single HRF at-risk alcohol use [38, 47–49].

### Sampling frame and participants

Over 17 consecutive months in 2011 and 2012, participants were recruited in the four major departments at the University Medicine Hospital Greifswald in Germany: internal medicine (endocrinology, nephrology, cardiology, gastroenterology, angiology, pneumology), general surgery (general and thorax surgery), trauma surgery, and ear-nose-throat. All wards of each department, except intensive care units, were included, resulting in 13 wards. The hospital provides general hospital care for 600,000 inhabitants in Greifswald and surrounding communities.

All patients aged 18–64 years admitted to one of the four departments were approached on weekdays, by one of three research assistants. Patients were asked to complete a self-administrated questionnaire on health behaviors using an electronic handheld device. Patients cognitively (*n* = 21) or physically incapable or terminally ill (*n* = 159), with highly infectious diseases (*n* = 101), discharged or transferred outside the study area within the first 24 h or within the weekend on which they were admitted (*n* = 627), already screened for the study during an earlier hospital stay (*n* = 2779), with insufficient German language skills (*n* = 93), or employed at the conducting research institute (*n* = 4) were excluded. In total, 6809 of 10,593 patients assessed met screening inclusion criteria. Of these, 414 were missed before discharge or transferal, 107 declined participation, 36 provided insufficient data, and 1 died, leaving 6251 (91.8%) participants who provided informed oral consent and sufficient data.

### Measurements

#### Behavioral HRFs

Four HRFs were assessed: two substance-use related HRFs (smoking, alcohol at-risk drinking) and two energy-balance related HRFs (physical inactivity, overweight). *Smoking* was assessed using the question “Are you a tobacco smoker currently?” and four response categories differentiating between current daily smoking, occasional smoking, former smoking and never smoking. Current occasional or daily smoking was considered as HRF. *Alcohol* at-risk drinking was determined using the total score of the Alcohol Use Disorder Identification Test-Consumption (range: 0–12) [50]. Alcohol at risk-drinking was considered present in females and males with scores of ≥4 and ≥ 5, respectively. These recommended gender-specific cut-offs had shown good sensitivity and specificity in detecting at-risk alcohol use including but not limited to alcohol use disorders [51], and correspond well to the national limits defined for healthy people, i.e. > 12/ 24 g of pure alcohol per day and > 3/ 4 drinks per occasion for women/ men [52]. *Overweight* was assessed using the body-mass-index obtained by self-reported weight in kilogram and height in meters. A body-mass-index ≥25.0 [53] was defined as overweight. Physical *inactivity* was measured by asking “Do you also do sports? “with six response categories: none, < 1, 1–2, 2–3, 3–4 and > 4 h per week. As at least 75 min of vigorous- or 150 min of moderate-intensity physical activity per week are recommended [54, 55], participants with none or less than 1 h were considered inactive.

#### Grouping variables

Medical department (internal medicine, general surgery, trauma surgery, earn-nose-throat) was recorded. Gender (male, female) was assessed. Participants were allocated to three age groups: 18–34, 35–49, 50–64 years. SES included school education and employment status. To determine highest school education achieved, various German school types were categorized as a) lowest level including 9 years of school or less, b) medium level including 10 to 11 years of school, and c) highest level including 12 or more years of school. Employment status distinguished between currently employed, unemployed, and other. “Other” included retired persons (69.2%), students (12.6%), housewives (6.1%), and not further specified (12.1%).

### Statistical analyses

Proportions and 95% confidence intervals (CI) for multiple, i.e. two or more HRFs, and for each of the 16 HRF profiles are given for the total sample; and separately for each medical department, gender, age group, level of school, and employment status. The 16 HRF profiles included one healthy profile (no HRF), four single factor profiles (*smoking*, *alcohol*, *overweight* or *inactivity* [only]), six profiles with two HRFs (*smoking plus alcohol*, *smoking plus overweight*, *smoking plus inactivity*, *alcohol plus overweight*, *alcohol plus inactivity*, *overweight plus inactivity*), four profiles with three HRFs (*smoking plus alcohol plus overweight*, *smoking plus alcohol plus inactivity*, *smoking plus overweight plus inactivity*, *alcohol plus overweight plus inactivity*), and one profile with all four HRFs (*smoking plus alcohol plus overweight plus inactivity*). Non-overlapping CIs were considered statistically significant, with two CIs just touching indicating significant differences at about *p* < 0.01 [56].

In addition, the mean number of HRFs and standard deviation (SD) are given for the total sample and separately for each subgroup. To investigate variables associated with HRF number (counts: 0, 1, 2, 3, 4), a multivariate poisson regression analysis with medical department, gender, age group, level of school, and employment status as predictors was calculated. *P*-values < 0.05 were considered statistically significant. Cases with missing values were excluded list-wise. STATA version 13.1 SE was used.

## Results

### Sample characteristics

The sample was on average 45.7 years old (SD = 13.3). With observed numbers given in Tables 1, 49.4% of the sample were aged 50 years or older, 58.6% were male, 21.8% had the lowest level of school education, 59.2% were employed, and 36.4% were recruited on the internal medicine department.

**Table 1:** Occurrence of each behavioral health risk factor in the total sample and stratified by medical department, gender, age and socio-economics

Subgroups	Number	Smoking		Alcohol		Inactivity		Overweight	
		*%*	*(95% CI)*	*%*	*(95% CI)*	*%*	*(95% CI)*	*%*	*(95% CI)*
Total	6251	39.0	(37.7–40.2)	21.2	(20.2–22.2)	65.6	(64.4–66.8)	61.0	(59.7–62.2)
Medical department									
Internal medicine	2284	33.8	(31.9–35.8)	21.2	(20.2–22.2)	71.7	(69.8–73.6)	64.6	(62.6–66.6)
General surgery	957	34.2	(31.1–37.3)	18.8	(17.2–20.5)	67.4	(64.3–70.4)	61.7	(58.5–64.7)
Ear-nose-throat	1282	44.1	(41.4–46.9)	23.2	(21.0–25.7)	64.4	(61.7–67.0)	56.7	(53.9–59.4)
Trauma surgery	1728	44.5	(42.1–46.9)	25.6	(23.5–27.7)	57.3	(55.0–59.7)	58.9	(56.5–61.2)
Gender									
Men	3665	42.6	(41.0–44.2)	27.9	(26.5–29.4)	65.3	(63.7–66.8)	66.0	(64.5–67.6)
Women	2586	33.8	(32.0–35.7)	11.7	(10.5–13.0)	66.0	(64.1–67.8)	53.8	(51.8–55.7)
Age in years									
18–34	1546	50.4	(47.9–52.9)	28.1	(25.9–30.5)	50.3	(47.7–52.8)	41.7	(39.2–44.2)
35–49	1615	43.9	(41.5–46.4)	22.2	(20.2–24.3)	68.5	(66.2–70.7)	63.3	(60.9–65.7)
50–64	3090	30.7	(29.0–32.2)	17.3	(16.0–18.7)	71.7	(70.1–73.3)	69.4	(67.7–71.0)
School education achieved									
Lowest level	1354	51.0	(48.3–53.7)	22.3	(20.1–24.6)	78.1	(75.8–80.3)	65.7	(63.1–68.2)
Medium level	3715	38.4	(36.8–39.9)	19.7	(18.5–21.1)	66.6	(65.1–68.1)	63.2	(61.6–64.8)
Highest level	1137	26.6	(24.0–29.2)	24.7	(22.2–27.3)	47.4	(44.5–50.4)	48.5	(45.5–51.4)
Employment									
Unemployed	749	52.2	(48.6–55.8)	28.8	(25.6–32.2)	75.7	(72.5–78.7)	62.8	(59.2–66.2)
Employed	3680	37.5	(35.9–39.0)	22.2	(20.8–23.5)	61.5	(59.9–63.0)	61.1	(59.5–62.6)
Other	1791	36.6	(34.4–38.9)	16.0	(14.3–17.7)	70.0	(67.8–72.1)	60.0	(57.7–62.3)

*Notes:* CI = confidence interval, non-overlapping 95% CIs indicate significant differences at *p* ≤ 0.01

### Single HRFs

Inactivity was the most common HRF (65.6%), followed by overweight (61.0%), smoking (39.0%), and at-risk alcohol use (henceforth alcohol, 21.2%). As indicated by non-overlapping 95% CIs in Table 1, statistically significantly differences in single HRFs were found across different medical departments, socio-demographic and socio-economic subgroups (*p* < 0.01). While a larger proportion of trauma surgery and in part ear-nose-throat patients reported smoking (44.5, 44.1% versus 33.8, 34.2%) and alcohol (25.6, 23.2% versus 21.2, 18.8%), a larger proportion of internal medicine and in part general surgery patients reported inactivity (71.7, 67.4% versus 57.3, 64.4%) and overweight (64.6, 61.7% versus 58.9, 56.7%, Table 1). Compared to women, a larger proportion of men reported smoking (42.6% versus 33.8%), alcohol (27.9% versus 11.7%) and overweight (66.0% versus 53.8%). The older the age group was, the lower was the proportion that reported smoking (50.4% versus 43.9% versus 30.7%) and alcohol (28.1% versus 22.2% versus 17.3%), and the higher the occurrence of inactivity (50.3% versus 68.5% versus 71.7%) and overweight (41.7% versus 63.3% versus 69.4%). The higher the level of school education was, the lower was the occurrence of smoking (51.0% versus 38.4% versus 26.6%), inactivity (78.1% versus 66.6% versus 47.4%) and/ or overweight (65.7, 63.2% versus 48.5%), but the occurrence of alcohol was higher in patients with the highest compared to those with medium level of school education (24.7% versus 19.7%). A larger proportion of unemployed patients reported smoking (48.6% versus 37.5, 36.6%), alcohol (28.8% versus 22.2, 16.0%) and inactivity (75.7% versus 61.5, 70.0%) than employed and other patients, respectively.

### HRF profiles and medical department

Overall, the mean HRF number was 1.9 (SD = 1.0, Table 2); 92.2% of the patients reported at least one and 65.7% multiple, i.e. two or more, HRFs. In the total sample and in eight of 15 subgroups, the three most common HRF profiles were *overweight plus inactivity* (22.4%), *overweight* (11.6%) and *smoking plus overweight plus inactivity* (10.7%). Having no HRF was ranked 6th among the 16 HRF profiles (7.8%).

**Table 2:** Occurrence of multiple health risk factors and of each behavioral health risk factor profile in the total sample and stratified by medical department

Health risk factor(s)	Total (*n* = 6251)		Internal medicine (*n* = 2284)		General surgery (*n* = 957)		Ear-nose-throat (*n* = 1282)		Trauma surgery (*n* = 1728)	
Mean number (standard deviation)	1.9	(1.0)	1.9	(0.9)	1.8	(0.9)	1.9	(1.0)	1.9	(1.0)
	*%*	*(95% CI)*	*%*	*(95% CI)*	*%*	*(95% CI)*	*%*	*(95% CI)*	*%*	*(95% CI)*
Multiple, ≥2	65.7	(64.5–66.9)	67.4	(65.5–69.3)	64.6	(61.5–67.6)	65.5	(62.8–68.1)	64.2	(61.9–66.5)
Profile										
no health risk factor	7.8	(7.1–8.5)	5.7	(4.8–6.7)	8.7	(7.0–10.6)	8.3	(6.9–10.0)	9.6	(8.3–11.1)
smoking (only)	3.7	(3.3–4.2)	2.4	(1.8–3.1)	3.2	(2.2–4.6)	4.7	(3.6–6.0)	5.0	(4.1–6.2)
alcohol (only)	1.4	(1.2–1.8)	0.8	(0.5–1.30)	1.6	(0.9–2.6)	1.8	(1.1–2.7)	1.9	(1.32–2.7)
overweight (only)	11.6	(10.8–12.4)	12.7	(11.4–14.2)	11.4	(9.4–13.6)	9.8	(8.3–11.6)	11.5	(10.0–13.1)
inactivity (only)	9.8	(9.0–10.5)	10.9	(9.7–12.3)	10.6	(8.7–12.7)	9.8	(8.3–11.6)	7.7	(6.5–9.1)
smoking *plus* alcohol	1.9	(1.6–2.3)	1.0	(0.6–1.51)	0.9	(0.4–1.8)	2.3	(1.52–3.2)	3.3	(2.5–4.3)
smoking *plus* overweight	4.0	(3.5–4.5)	2.8	(2.1–3.5)	3.6	(2.5–4.9)	4.4	(3.4–5.7)	5.6	(4.6–6.8)
smoking *plus* inactivity	8.8	(8.1–9.5)	8.7	(7.5–9.9)	8.5	(6.8–10.4)	10.5	(8.9–12.3)	7.9	(6.6–9.2)
alcohol *plus* overweight	2.5	(2.1–2.9)	2.1	(1.6–2.8)	2.0	(1.2–3.1)	2.5	(1.7–3.5)	3.2	(2.4–4.1)
alcohol *plus* inactivity	1.4	(1.1–1.7)	1.6	(1.1–2.2)	1.8	(1.0–2.8)	1.2	(0.7–2.0)	1.0	(0.6–1.6)
overweight *plus* inactivity	22.4	(21.4–23.4)	27.6	(25.8–29.5)	27.0	(24.2–29.9)	18.2	(16.1–20.4)	16.0	(14.3–17.8)
smoking *plus* alcohol *plus* overweight	1.5	(1.2–1.9)	0.7	(0.4–1.2)	1.3	(0.6–2.2)	1.8	(1.1–2.7)	2.5	(1.8–3.3)
smoking *plus* alcohol *plus* inactivity	4.3	(3.8–4.8)	4.3	(3.5–5.2)	3.1	(2.1–4.4)	4.6	(3.5–5.9)	4.7	(3.8–5.9)
smoking *plus* overweight *plus* inactivity	10.7	(10.0–11.5)	10.4	(9.2–11.7)	10.8	(8.9–12.9)	10.9	(9.2–12.8)	11.1	(9.6–12.6)
alcohol *plus* overweight *plus* inactivity	4.2	(3.8–4.8)	4.6	(3.8–5.5)	2.9	(2.0–4.2)	4.1	(3.1–5.4)	4.6	(3.6–5.7)
all four health risk factors	4.0	(3.5–4.5)	3.6	(2.9–4.5)	2.8	(1.9–4.1)	4.9	(3.8–6.2)	4.4	(3.5–5.5)

*Notes:* CI = confidence interval, non-overlapping 95% CIs indicate significant differences at p ≤ 0.01

Patients from different medical departments did not differ significantly concerning the occurrence of any and multiple HRFs (Table 2). Concerning the HRF profiles, five HRF profiles, each involving smoking and/ or alcohol were significantly more common in trauma surgery (*smoking*, *alcohol*, *smoking plus alcohol*, *smoking plus overweight*, *smoking plus alcohol plus overweight*) and partly in ear-nose-throat patients (*smoking*, *alcohol*, *smoking plus alcohol*) than in internal medicine patients (Table 2). *Smoking plus alcohol* was also more common in trauma surgery patients than in general surgery patients. Two energy-balance related HRF profiles were significantly more common in internal medicine than in trauma surgery patients *(inactivity),* and in internal medicine and general surgery patients than in ear-nose-throat and trauma surgery patients *(overweight plus inactivity)*.

### HRF profiles, gender and age

A significantly larger proportion of men reported any and multiple (71.2% versus 57.9%) HRFs than women (Table 3). Six of the 15 risky HRF profiles, each with multiple HRFs and alcohol *(smoking plus alcohol, alcohol plus overweight, smoking plus alcohol plus overweight, smoking plus alcohol plus inactivity, alcohol plus overweight plus inactivity*, and all four HRFs), were significantly more common in men. Three frequent HRF profiles, each involving inactivity and a maximum of two HRFs (*inactivity, smoking plus inactivity, overweight plus inactivity)* were more common in women.

**Table 3:** Occurrence of multiple health risk factors and of each behavioral health risk factor profile stratified by gender and age

Health risk factor(s)	Women (*n* = 2586)		Men (*n* = 3665)		18–34 years (*n* = 1546)		35–49 years (*n* = 1615)		50–64 years (*n* = 3090)	
Mean number (standard deviation)	1.7	(0.9)	2.0	(1.0)	1.7	(1.0)	2.0	(1.0)	1.9	(0.9)
	*%*	*(95% CI)*	*%*	*(95% CI)*	*%*	*(95% CI)*	*%*	*(95% CI)*	*%*	*(95% CI)*
Multiple, ≥2	57.9	(56.0–59.8)	71.2	(69.7–72.7)	57.3	(54.8–59.8)	69.5	(67.2–71.8)	67.9	(66.3–69.6)
Profile										
no health risk factor	10.9	(9.7–12.2)	5.6	(4.8–6.4)	12.8	(11.2–14.6)	6.7	(5.6–8.1)	5.8	(5.0–6.7)
moking (only)	4.0	(3.3–4.8)	3.5	(2.9–4.2)	8.2	(6.9–9.7)	3.1	(2.30–4.1)	1.8	(1.3–2.31)
alcohol (only)	1.4	(0.9–1.9)	1.5	(1.1–1.9)	3.9	(3.0–5.0)	0.9	(0.5–1.5)	0.5	(0.3–0.8)
overweight (only)	11.3	(10.1–12.6)	11.8	(10.8–12.9)	8.3	(7.0–9.8)	9.9	(8.5–11.5)	14.1	(12.9–15.4)
inactivity (only)	14.5	(13.2–16.0)	6.4	(5.6–7.2)	9.4	(8.0–11.0)	9.8	(8.4–11.4)	9.9	(8.8–11.0)
smoking *plus* alcohol	1.0	(0.7–1.5)	2.5	(2.0–3.1)	5.3	(4.2–6.5)	1.2	(0.76–1.9)	0.5	(0.3–0.84)
smoking *plus* overweight	3.3	(2.6–4.0)	4.5	(3.9–5.3)	5.8	(4.6–7.0)	5.3	(4.3–6.5)	2.5	(1.9–3.1)
smoking *plus* inactivity	10.2	(9.0–11.4)	7.8	(7.0–8.7)	10.9	(9.4–12.6)	8.7	(7.3–10.1)	7.8	(6.9–8.8)
alcohol *plus* overweight	1.5	(1.0–2.0)	3.2	(2.6–3.8)	2.7	(1.9–3.6)	2.6	(1.9–3.5)	2.3	(1.8–2.9)
alcohol *plus* inactivity	1.0	(0.7–1.5)	1.6	(1.2–2.1)	1.8	(1.21–2.6)	0.7	(0.3–1.22)	1.5	(1.1–2.0)
overweight *plus* inactivity	24.3	(22.6–26.0)	21.0	(19.7–22.4)	8.8	(7.4–10.3)	20.9	(18.9–22.9)	30.0	(28.4–31.6)
smoking *plus* alcohol *plus* overweight	0.7	(0.4–1.1)	2.1	(1.7–2.6)	2.8	(2.0–3.7)	1.7	(1.16–2.5)	0.8	(0.5–1.15)
smoking *plus* alcohol *plus* inactivity	3.2	(2.6–4.0)	5.0	(4.4–5.8)	6.0	(4.8–7.2)	5.5	(4.4–6.7)	2.8	(2.3–3.5)
smoking *plus* overweight *plus* inactivity	9.8	(8.7–11.0)	11.4	(10.4–12.5)	7.6	(6.3–9.0)	13.4	(11.8–15.1)	10.9	(9.9–12.1)
alcohol *plus* overweight *plus* inactivity	1.3	(0.9–1.8)	6.3	(5.6–7.2)	1.9	(1.3–2.7)	4.6	(3.6–5.7)	5.2	(4.5–6.1)
all four health risk factors	1.7	(1.2–2.2)	5.6	(4.9–6.4)	3.9	(3.0–5.0)	5.0	(3.9–6.1)	3.5	(2.9–4.2)

*Notes:* CI = confidence interval, non-overlapping 95% CIs indicate significant differences at p ≤ 0.01

A significantly larger proportion of both older age groups (i.e. 35–49 and 50–64 year olds) reported any and multiple (69.5, 67.9% versus 57.3%) HRFs than the youngest age group (18–35 year olds, Table 3). While having no HRF was the most common profile in the youngest age group (12.8%), seven of the 15 risky HRF profiles, each involving smoking and/ or alcohol, were significantly more common in either the youngest (*smoking, alcohol, smoking plus alcohol, smoking plus inactivity*) or in both younger age groups, i.e. the 18–34 and 35–49 year olds (*smoking plus overweight, smoking plus alcohol plus overweight, smoking plus alcohol plus inactivity*). Four HRF profiles, each involving overweight, were significantly more common in either the oldest compared to both younger groups (*overweight*) or among both older groups compared to the youngest (*smoking plus overweight plus inactivity, alcohol plus overweight plus inactivity*). The occurrence of the most common HRF profile *overweight plus inactivity* increased significantly across age groups (8.8% versus 20.9% versus 30.0%).

### HRF profiles, school education and employment status

The lower the level of school was, the significantly larger the proportion of any and multiple HRFs (79.0% versus 66.5% versus 48.0%, Table 4). While having no HRF was the most common profile in the group with the highest level of school (16.9%), six of the 15 risky HRF profiles, each involving a single HRF or two HRFs including alcohol, were also significantly more common in groups with higher school levels: either in the group with the highest level compared to the two groups with lower levels (*alcohol, smoking plus alcohol*), in the two groups with higher levels compared to the group with the lowest level (*overweight, inactivity*), or in the group with the highest level compared to the group with the lowest level *(alcohol plus overweight, alcohol plus inactivity).* Five HRF profiles, each involving two or more HRFs including inactivity*,* were significantly more common in groups with lower school level: either in the group with the lowest level compared to the two groups with higher levels (*smoking plus alcohol plus inactivity*), in the two groups with lower levels compared to the group with the highest level (*overweight plus inactivity*), or occurrence decreased significantly with each level of school (*smoking plus inactivity, smoking plus overweight plus inactivity,* and all four HRFs).

**Table 4:** Occurrence of multiple health risk factors and of each behavioral health risk factor profile stratified by school education (lowest, medium, highest) and employment status

Health risk factor(s)	Lowest (*n* = 1354)		Medium (*n* = 3715)		Highest (*n* = 1137)		Unemployed (*n* = 749)		Employed (*n* = 3680)		Others (*n* = 1791)	
Mean number (standard deviation)	2.2	(0.9)	1.9	(0.9)	1.5	(1.0)	2.2	(0.9)	1.8	(1.0)	1.8	(0.9)
	*%*	*(95% CI)*	*%*	*(95% CI)*	*%*	*(95% CI)*	*%*	*(95% CI)*	*%*	*(95% CI)*	*%*	*(95% CI)*
Multiple, ≥2	79.0	(76.8–81.2)	66.5	(64.9–68.0)	48.0	(45.1–51.0)	77.8	(74.7–80.8)	63.6	(62.0–65.1)	65.3	(63.1–67.5)
Profile												
no health risk factor	2.3	(1.6–3.2)	7.0	(6.2–7.8)	16.9	(14.8–19.2)	2.1	(1.2–3.4)	9.3	(8.4–10.3)	6.9	(5.7–8.1)
smoking (only)	3.7	(2.8–4.8)	3.6	(3.0–4.3)	4.0	(3.0–5.4)	2.8	(1.7–4.3)	4.0	(3.4–4.6)	3.6	(2.8–4.6)
alcohol (only)	0.5	(0.2–1.1)	0.8	(0.5–1.2)	4.7	(3.5–6.1)	0.9	(0.4–1.9)	1.7	(1.3–2.2)	1.2	(0.7–1.8)
overweight (only)	7.3	(6.0–8.8)	12.3	(11.2–13.4)	14.2	(12.2–16.3)	8.5	(6.6–10.8)	12.5	(11.5–13.6)	10.7	(9.3–12.2)
inactivity (only)	7.2	(5.8–8.7)	9.9	(8.9–10.9)	12.2	(10.4–14.3)	7.7	(5.9–9.9)	8.9	(8.0–9.9)	12.3	(10.9–14.0)
smoking *plus* alcohol	1.7	(1.1–2.5)	1.2	(0.9–1.6)	4.4	(3.3–5.8)	2.4	(1.4–3.8)	1.8	(1.4–2.3)	1.8	(1.2–2.5)
smoking *plus* overweight	3.2	(2.4–4.3)	4.6	(3.9–5.3)	3.1	(2.2–4.3)	3.9	(2.6–5.5)	4.5	(3.86–5.2)	3.0	(2.3–3.92)
smoking *plus* inactivity	11.9	(10.2–13.7)	9.0	(8.1–10.0)	4.3	(3.2–5.7)	10.8	(8.7–13.3)	8.1	(7.2–9.0)	9.5	(8.2–10.9)
alcohol *plus* overweight	1.5	(0.9–2.3)	2.5	(2.0–3.1)	3.7	(2.7–5.0)	1.9	(1.0–3.1)	2.9	(2.4–3.5)	1.8	(1.3–2.6)
alcohol *plus* inactivity	0.8	(0.4–1.4)	1.3	(1.0–1.7)	2.3	(1.5–3.3)	1.6	(0.8–2.8)	1.5	(1.2–2.0)	0.9	(0.5–1.4)
overweight *plus* inactivity	25.3	(23.0–27.7)	23.3	(22.0–24.7)	16.3	(14.2–18.5)	20.4	(17.6–23.5)	20.8	(19.5–22.2)	26.7	(24.7–28.9)
smoking *plus* alcohol *plus* overweight	1.6	(1.0–2.4)	1.5	(1.1–1.9)	1.7	(1.0–2.6)	1.7	(0.9–2.9)	1.7	(1.3–2.2)	1.1	(0.6–1.7)
smoking *plus* alcohol *plus* inactivity	6.3	(5.0–7.7)	4.0	(3.4–4.7)	2.7	(1.9–3.8)	8.8	(6.9–11.1)	3.6	(3.0–4.2)	3.8	(3.0–4.8)
smoking *plus* overweight *plus* inactivity	16.8	(14.8–18.9)	10.6	(9.6–11.6)	4.3	(3.2–5.7)	14.8	(12.4–17.6)	9.7	(8.8–10.7)	11.3	(9.9–12.8)
alcohol *plus* overweight *plus* inactivity	4.1	(3.1–5.3)	4.6	(3.9–5.3)	3.3	(2.3–4.5)	4.5	(3.2–6.3)	4.8	(4.1–5.6)	2.8	(2.1–3.7)
all four health risk factors	5.8	(4.6–7.2)	3.9	(3.3–4.5)	2.0	(1.3–3.0)	6.9	(5.2–9.0)	4.0	(3.4–4.7)	2.6	(1.9–3.4)

*Notes:* CI = confidence interval, non-overlapping 95% CIs indicate significant differences at p ≤ 0.01

A significantly larger proportion of unemployed patients reported any and multiple (77.8% versus 63.6, 65.3%) HRFs than employed and other patients (Table 4). Three HRF profiles, each involving three or all four HRFs and smoking were significantly more common in unemployed than in employed (*smoking plus alcohol plus inactivity, smoking plus overweight plus inactivity*, all four HRFs) and other patients (*smoking plus alcohol plus inactivity,* all four HRFs). Two HRF profiles were significantly more common in employed than in unemployed (*overweight*) or other patients (*alcohol plus overweight plus inactivity*). Two HRF profiles (*inactivity*, *overweight plus inactivity)* were significantly more common in others than in the employed and unemployed.

### Multivariate analysis on the number of HRFs

As depicted in Table 5, the multivariate analysis revealed that the number of HRFs was significantly increased in males (incident rate ratio, IRR = 1.18), 35–49 year olds (IRR = 1.13), 50–64 year olds (IRR = 1.07), persons with the lowest (IRR = 1.39) or medium (IRR = 1.23) level of school education, and unemployed persons (IRR = 1.12). No significantly increased IRRs were found for medical department.

**Table 5:** Multivariate poisson regression analysis predicting the number of reported health risk factors (*n* = 6205)

Predictors		incident rate ratio	95% CI	p
Medical department (internal medicine*)	General surgery	0.99	0.93–1.04	0.638
	Ear-nose-throat	1.02	0.97–1.07	0.430
	Trauma surgery	1.01	0.96–1.06	0.655
Gender (female*)	Male	1.18	1.14–1.23	< 0.001
Age group (18–34 years*)	35–49 years	1.13	1.07–1.19	< 0.001
	50–64 years	1.07	1.02–1.12	0.01
Achieved school level (highest*)	Lowest	1.39	1.31–1.48	< 0.001
	Medium	1.23	1.16–1.30	< 0.001
Employment status (employed*)	Unemployed	1.12	1.06–1.19	< 0.001
	Other	0.99	0.95–1.04	0.677

*Notes:* * reference, CI = confidence interval

## Discussion

So far, little is known about HRF profiles in general hospital patients, and this study helps to fill an important gap of knowledge. Five key findings emerged from the study: 1) Regardless of medical department, two thirds of all patients reported multiple behavioral HRFs. 2) While overall the most common HRF profiles involved energy-balance behaviors, trauma surgery and ear-nose-throat patients had particularly increased rates of substance-use related HRF profiles. 3) Men had higher rates than women concerning almost all HRF profiles involving multiple HRFs and alcohol. 4) While older patients had higher rates of HRF profiles involving multiple and both energy-balance related HRFs, younger patients had higher rates of HRF profiles involving at least one substance-use related HRF. 5) A social gradient was found: The lower the school education, the riskier the HRF profiles.

Compared to the general population, the study revealed similar proportions of the single HRFs alcohol, overweight and physical inactivity among general hospital patients [11]. However, markedly larger proportions of current smokers (39% versus 28%) and of persons with two or more HRFs were found (66% versus 55%, 11) indicating that needs in terms of behavior change interventions are particularly complex in general hospital patients. This appears to be plausible as hospital patients may have been admitted for reasons attributable to HRFs [1, 2], and as co-occurring HRFs can have more than additive effects on disease incidence (e.g. 17, 18). However, in addition to the fact that general hospital patients may be considered to be more morbid than the general population, the differences and similarities noted may be under- or overestimated due to different sample characteristics resulting from different inclusion criteria. For example, this study’s sample that included adult patients aged 18–64 years regardless of living situation, was younger (mean age of 46 versus 60 years), included fewer females (41% versus 52%), and fewer persons with the lowest level of school (22% versus 32%) than the German general population sample that included adults living in a private household with landline phone numbers regardless of age [11]. Nevertheless, the large proportion of general hospital patients with two or more HRFs shows a particular high need of preventive measures simultaneously targeting multiple HRFs in the general hospital setting.

Energy-balance related HRF profiles were particularly common in the total sample. With a proportion of 22%, *overweight plus inactivity* was the most common HRF profile as also found in a general population and in a general hospital study in Germany [11, 44]. Similar to a previous general hospital study that investigated the HRFs smoking and alcohol only [57], our findings showed that about half of the hospital patients reported at least one of the two substance-use related HRFs. The current study also revealed that the occurrence of energy-balance HRF profiles was even larger in internal medicine and general surgery patients, while substance-use related HRF profiles were more common in ear-nose-throat and trauma surgery patients. The different relevance of various HRF profiles in different medical departments is plausible. For example, joint at-risk alcohol use and tobacco smoking has more than a multiplicative effect on risk of head and neck cancers [18] which are typically treated in ear-nose-throat departments. Although differences in proportional relevance need to be considered when providing preventive measures, our findings also indicate that they should not be limited to single HRFs or HRF profiles as all four HRFs were common in all departments investigated.

In line with previous studies, gender, age and socio-economic subgroups differed concerning the occurrence of any and multiple HRFs, but also concerning single HRF profiles (e.g. 11, 19). Among male, older, lower educated, and unemployed patients, larger proportions of any and multiple HRFs were found compared to their respective counterparts. The magnitude of difference was particularly large concerning education: 79% of the patients with the lowest versus 48% of the patients with the highest level of school reported multiple HRFs. There was a clear social gradient concerning both SES-related measures: The lower the level of school was, the higher were the proportions of patients with any HRFs, two or more HRFs, and all four HRFs. In addition, unemployment was also a significant and independent predictor of the accumulation of HRFs. Recent studies have shown that social inequalities with regards to the accumulation of HRFs have rather increased over the years [58]. This may also explain the increased social disparities in adult mortality in some regions of developed countries [59]. Our findings support the necessity of behavior change interventions to aim at closing the gap or (at least) at preventing a further widening of the gap by achieving greater reach and greater effectiveness in low SES groups [60]. For example, given that duration of unemployment has been shown to be related to the accumulation of HRFs [61], providing screening and brief intervention at job-agencies could be a feasible and effective approach as found concerning reduced alcohol use [62].

In addition to subgroup differences concerning the number of HRFs, subgroups also differed concerning HRF profiles. Overall, female, older, lower educated, and employed patients tended to show energy-balance related HRF profiles; and male, younger, higher educated, and unemployed patients tended to show substance-use related HRF profiles more often than their respective counterparts. However, while in some subgroup comparisons, profiles involving certain HRFs occurred more frequently in one group, riskier profiles involving the same HRF occurred more frequently in the comparison group. In particular, the rates of HRF profiles involving at-risk alcohol use were higher in patients with the highest compared to those with the lowest level of school. This fits in with findings from general population studies revealing higher proportions of alcohol drinkers and higher consumption scores with higher education and higher SES (e.g. [63, 64]). However, also in line with previous research [19], the patients with the lowest level of school more often reported riskier HRF profiles involving alcohol, namely profiles that involved multiple other HRFs in addition to at-risk alcohol use. Similarly, women clearly showed larger proportions of three of the most common HRF profiles, all including inactivity with a maximum of one more HRF. However, in line with previous findings [19], men more often reported those HRF profiles that included inactivity plus two or three more HRFs. Brief interventions targeting multiple HRFs need to consider this.

The strengths of this study include: The sample included a large proportion of all eligible patients (92%). It may also be considered representative of general hospital patients treated for various diseases and injuries at four major general hospital departments. We investigated not only the occurrence but also the co-occurrence of four behavioral HRFs known as major contributors to the development and maintenance of non-communicable diseases in general hospital patients. Our study delineates differences in HRFs between different disciplines treating patients. It investigated unemployment as an independent predictor of the accumulation of HRFs in addition to school education.

Several limitations of the study need to be considered. The first limitation concerns the possible underestimation of the proportions of HRFs. The assessment was based on self-report and might be distorted by patients’ tendencies to provide socially desirable answers. For example, self-reported statements as used in our study are likely to underestimate overweight [65]. The inactivity measure used may have underestimated either physical inactivity or physical activity. Although HRF definitions were based on current recommendations [2, 6], the measure provided a rather rough and limited estimation of physical activity. For example, it assessed sports activity only rather than in combination with job-, transport- and housework-related physical activity as done by the international physical activity questionnaire [66]; and it did not differentiate intensity levels of physical activity for which separate recommendations exist, e.g. at least 75 min of vigorous- or 150 min of moderate-intensity physical activity per week [54, 55]. Due to item restrictions, and to prevent misclassification of patients with vigorous-intensity activity, a more liberal cut-off than recommended, i.e. less than 1 hour of sports per week was applied to determine physical inactivity. Given that over-reporting of physical activity is a common problem, also when assessed by more established questionnaires [67], and as physical activity was not in the focus of the original trial which focused on alcohol [49], a simple measure was preferred to keep the screening as brief as possible. Furthermore, HRFs on general surgery wards may have been underestimated as many surgical patients present on internal medicine wards first, and this study only assessed data during their first hospital admission. The second limitation concerns the generalizability of our findings to patients from other departments, or to other general hospitals in Germany or beyond. A multi-site study in Germany, however, revealed similar proportions of the single HRFs [44]. The third limitation concerns that some significant differences in proportions between subgroups may have been missed. By interpreting non-overlapping 95% confidence intervals (a common procedure in epidemiological research), differences at about *p* = 0.01 and smaller may be found, but differences at about *p* > 0.01 to *p* < 0.05 may remain undetected [56]. However, to avoid multiple testing and inflation of *p*-values, additional test statistics were not calculated in this study. The final limitation concerns the lack of information on the medical condition, costs of treatment and length of hospital stay. It may be expected that the co-occurrence of HRFs and the occurrence of particular HRF profiles may be related to higher health care costs. Future research should investigate this.

## Conclusions

Given that a lower number of behavioral HRFs is related to a lower risk of mortality [15, 16], and two thirds of the patients reporting profiles involving multiple HRFs, our study showed a high need for systematic screening and intervention targeting multiple HRF in general hospital patients. As health care still lacks systematic preventive measures accessible to all patients, the potential for the prevention and treatment of non-communicable diseases is being missed.

Systematically identifying and addressing patients’ individual HRF profiles may have a great potential for at least two reasons: Firstly, it may simultaneously serve primary and secondary prevention purposes by preventing the onset of chronic diseases in patients who have not been diagnosed with chronic diseases yet, and by improving treatment success and prognosis in patients with chronic diseases. Although a large part of the general hospital patients is expected to have been diagnosed with chronic diseases in the past, our study supports previous findings showing that routine health care alone may not be sufficient for patients with chronic diseases to change their lifestyle [68, 69]. Secondly, systematic multiple HRF screening and intervention may provide the means to reach most patients, including those most in need, namely male, older, lower educated, and unemployed patients as indicated by our findings. These subgroups, and low SES people in particular have been found to be particularly hard to reach otherwise [70].

Medical care staff or health behavior change interventionists should be prepared to screen for and address multiple HRFs in each patient. With high reach of a systematic multiple HRF screening and efficacy of single HRF interventions [29–37], a multiple HRF approach is likely to have clinical and public health impact [71, 72]. Future research and implementation research in particular should investigate this.

## Data Availability

The datasets generated and analyzed during the current study are not publicly available due to the German data protection law but are available from the corresponding author on reasonable request that complies with the study purpose and the participants informed consent.

## Abbreviations

CI: Confidence interval
HRF: Health risk factor
M: Mean
PECO: Randomized controlled trial “Testing delivery channels of individualized motivationally tailored alcohol interventions among general hospital patients: in-person versus computer-based”
SD: Standard deviation
SES: Socio-economic status
